# Discovery of Potential Inhibitors of SARS-CoV-2 Main Protease by a Transfer Learning Method

**DOI:** 10.3390/v15040891

**Published:** 2023-03-30

**Authors:** Huijun Zhang, Boqiang Liang, Xiaohong Sang, Jing An, Ziwei Huang

**Affiliations:** 1 Cechanover Institute of Precision and Regenerative Medicine, School of Medicine, The Chinese University of Hong Kong (Shenzhen), Shenzhen 518172, China; 2 School of Life Sciences, University of Science and Technology of China, Hefei 230026, China; 3 Nobel Institute of Biomedicine, Zhuhai 519080, China; 4 Division of Infectious Diseases and Global Public Health, Department of Medicine, School of Medicine, University of California San Diego, La Jolla, CA 92093, USA; 5 School of Life Sciences, Tsinghua University, Beijing 100084, China

**Keywords:** deep learning, SARS-CoV-2 M^pro^, transfer learning, drug development, natural compound

## Abstract

The COVID-19 pandemic caused by SARS-CoV-2 remains a global public health threat and has prompted the development of antiviral therapies. Artificial intelligence may be one of the strategies to facilitate drug development for emerging and re-emerging diseases. The main protease (M^pro^) of SARS-CoV-2 is an attractive drug target due to its essential role in the virus life cycle and high conservation among SARS-CoVs. In this study, we used a data augmentation method to boost transfer learning model performance in screening for potential inhibitors of SARS-CoV-2 M^pro^. This method appeared to outperform graph convolution neural network, random forest and Chemprop on an external test set. The fine-tuned model was used to screen for a natural compound library and a *de novo* generated compound library. By combination with other in silico analysis methods, a total of 27 compounds were selected for experimental validation of anti-M^pro^ activities. Among all the selected hits, two compounds (gyssypol acetic acid and hyperoside) displayed inhibitory effects against M^pro^ with IC50 values of 67.6 μM and 235.8 μM, respectively. The results obtained in this study may suggest an effective strategy of discovering potential therapeutic leads for SARS-CoV-2 and other coronaviruses.

## 1. Introduction

SARS-CoV-2, first reported in the beginning of 2020 [1], has caused over 759 million confirmed infection cases including 6.8 million deaths as of March of 2023 as reported to the World Health Organization (WHO). SARS-CoV-2 is a novel coronavirus which shares 79.5% sequence similarity with SARS-CoV [2], both of which belong to the Coronaviridae family, which contains positive single-stranded encapsulated viruses [3]. The virus genome contains several open-reading frames (ORFs) that encode four structure proteins (sps), 16 non-structure proteins (nsps) and several accessory proteins [4,5]. Nsp5 is the main protease (M^pro^), which is also known as 3-Chymotrypsin like protease (3CL^pro^). It has been characterized as one of the potential druggable targets of SARS-CoV-2 owing to its essential role in viral replication and transcription [6]. Active M^pro^ consists of a homodimer while each protomer has three domains (I–III) [7]. The active site of M^pro^ locates in the cleft between domains I and II and features the catalytic Cys-His dyad (Cys145-His41) [8,9,10]. After ORF1a/b translates into two polyproteins pp1a and pp1ab, M^pro^ cleavages at 11 distinct sites to release functional polypeptides [6,11,12]. The core recognition sequence is Leu-Gln↓ (Ser/Ala/Gly) [7,13]. Moreover, the high conservatism of M^pro^ among coronaviruses and the absence of homologues with similar cleavage specificity in humans make it an attractive target for antiviral drug discovery [14,15].

Many clinical trials have been initiated in the search for the prevention and treatment of coronavirus disease 2019 (COVID-19). At the time of writing, several vaccines have been approved by the U.S. Food and Drug Administration (FDA), including ones by Pfizer/BioNTech, Moderna and Johnson and Johnson/Jassen (JnJ) [16]. There have also been attempts in preclinical development of multiple formulations of vaccine candidates [17]. However, the continuing mutations in the viral genome may affect the protective effects of current vaccines. Notably, the emergence of the Omicron (B.1.1.529) VoC which contains a high number of mutations in the viral spike protein has an increased reinfection risk [18]. As the pandemic threat continues and vaccines cannot provide complete and lasting protection [19], the need for antiviral agents to treat infected patients remains. Drug repurposing, for the advantage of already confirmed clinical profiles data, is considered to be a fast and low-cost approach to find potential effective therapeutic agents against COVID-19 [20,21,22]. At present, there are only three drugs approved by the FDA for the treatment of COVID-19, including Actemra (Tocilizumab), Veklury (Remdesivir) and Olumiant (baricitinib) [23]. There are several authorized products under an EUA for the clinical treatment of COVID-19 as well, including two anti-viral drugs which are Paxlovid (nirmatrelvir and ritonavir) and Lagevrio (molnupiravir), three immune modulators, five SARS-CoV-2-targeting monoclonal antibodies, sedatives and renal replacement therapies. Hundreds of drugs are undergoing clinical trials for COVID-19, such as favipiravir, lopinavir, ribavirin, ritonavir, and tocilizumab, which have shown positive effects in vitro [17,24]. Dexamethasone and hydroxychloroquine have been withdrawn from treatment options because of the insignificant protection benefits and serious side effects [24,25,26].

Drug discovery and development is a time-consuming process in which computational methods can help speed up the identification and application of drug candidates. Deep learning techniques have recently received wide attention and been applied to drug discovery [27]. To facilitate efforts in exploring the chemical space against various therapeutic targets for SARS-CoV-2, deep learning combined with computer-aided drug design (CADD) methodologies such as docking and molecular dynamics simulation have been extensively used [20,28,29,30,31,32,33,34,35]. However, labeled data scarcity remains a challenge for supervised learning due to time-consuming and laborious benchwork testing. To better solve this problem, transformer pre-training by making use of large amounts of unlabeled data plus downstream task-specific finetuning has become a powerful architecture for learning representation of texts, i.e., natural language processing (NLP) [36,37,38,39,40]. Compared with many previous approaches such as graph neural networks (GNNs), modern transformers display substantial gain of efficiency and throughput [41,42]. Given the availability of millions of Simplified Molecular-Input Line-Entry system (SMILES) strings, different molecular property prediction tasks can be tackled by using learned representations of functional groups and atoms learned by the model [43,44,45].

In the present study, we used pre-trained ChemBERTa [39] which is based on RoBERTa [37] transformer implementation from HuggingFace and fine-tuned it on a dataset which contains over 280,000 molecules screened against SARS-CoV-1 M^pro^ [29]. Considering the fact that natural compounds have been sources of pharmacologically active molecules for a long history and that the *de novo* design of novel scaffolds might expand the chemical space of active drug candidates, we made predictions of two libraries, a natural compound library (TargetMol) and a *de novo* generated compound library from the literature by Santana et al. [29], to seek molecules against SARS-CoV-2 M^pro^. The predicted active molecules were evaluated using molecular docking and PAINS filtering. In vitro enzyme activity inhibition experiments were performed to validate the selected hits.

## 2. Materials and Methods

### 2.1. Dataset Preparation

Due to the high sequence similarity (~76%) shared between SARS-CoV-2 M^pro^ and SARS-CoV-1 M^pro^, we selected a dataset which contains over 280,000 molecules against SARS-CoV-1 M^pro^ as the fine-tuning dataset. Obtained from the publication of Santana and Silva-Jr [29], it consisted of 629 active molecules and 288,940 inactive molecules. Based on the fact that one molecule can be represented by more than one SMILES strings, and that the augmented dataset with enumerated SMILES could help improve model performance [46], we used the same approach to augment the dataset. Different ratios of SMILES enumeration were calculated with a python script, which is available at https://github.com/Ebjerrum/SMILES-enumeration (accessed on 1 July 2020).

### 2.2. Chemical Space Analysis

Morgan fingerprints for each molecule using radius 2 and 2048 bits fingerprint vectors were determined after obtaining the canonical SMILES by rdkit in Python. Then, t-Distributed Stochastic Neighbor Embedding (t-SNE) clustering analysis was performed by the scikit-learn package in Python. Data points were reduced from 2048 dimensions to 2 dimensions by t-SNE. All t-SNE parameters were Scikit-learn’s default values.

### 2.3. Model Performance Evaluation

The fine-tuned model performance was evaluated with five-fold cross-validation. Scaffold splitting was used to ensure that the training/validation set is more structurally different, which, as a result, is more challenging for the model. Additionally, an external independent test dataset which was collected from results of a screening assay against SARS-CoV-2 M^pro^ using X-ray crystallography (at Diamond Light Source, Oxfordshire, United Kingdom) [47] was used. It consisted of 880 molecules with 78 hits. The performance of Chemprop [48], which is a freely available message passing neural network (MPNN) (http://chemprop.csail.mit.edu/predict (accessed on 27 October 2021)) on the same dataset, was also determined for comparison. Various evaluation metrics including area under the receiver–operator characteristic curve (au_roc), area under the precision–recall curve (au_prc), recall score, accuracy score, precision score and f1 score were calculated. Recall = TP/(TP + FN). Accuracy = (TP + TN)/(TP + FN + TN + FP). Precision = TP/(TP + FN). F1 = 2 × recision × Recall/(Precision + Recall). TP, TN, FP and FN stand for true positive, true negative, false positive and false negative, respectively. Figures were plotted by matplotlib in Python.

### 2.4. Compound Libraries and Compounds

The Natural Compound Library obtained from Targetmol (L6000) contains 2364 compounds after 228 compounds with large molecular weight were removed. The *de novo* generated compound library of Santana and Silva-Jr contains 66,392 generated molecules. PF-07321332 and Boceprevir were purchased from MedChemExpress. Compounds T2983, T3872, T2765, T2950, T2730, T2755, T2957, T3012, T2133, T3227, T1016, T2844, T2775, T1648, T1400, T1160, T2570, T3232, T5429, T2727, T5497, T1035, T1609, T6S1529, T3149, T3S1612 and TL0006 were purchased from TargetMol.

### 2.5. PAINS Filtering

All predicted active compounds were submitted to FAF-DRUGS4 server (available at http://fafdrugs4.mti.univ-paris-diderot.fr (accessed on 25 November 2021)) by evaluating their physicochemical properties [49]. Molecules with suspicious substructure features were flagged out by Pan Assay Interference Compounds (PAINS) filter.

### 2.6. Molecular Docking Protocol

Crystal structures of SARS-CoV-2 M^pro^ bound with inhibitor PF-07321332 (PDB ID: 7VH8) and inhibitor N3 (PDB ID: 6LU7) were accessed from the RCSB Protein Data Bank. The M^pro^ protein and inhibitor ligands were prepared using AutoDockTools by removing water atoms and adding polar hydrogen atoms and charges. Prepared protein and ligand files were converted to PDBQT format. Molecular docking was carried out using AutoDock Vina-1.2.0 software while M^pro^ in the structure of 7VH8 was used as the docking protein due to its higher resolution. The redocking of PF-07321332 and N3 was performed in order to validate the performance of the docking model; then, the docking model was determined for the virtual screening process. The grid box center was set at X: −18.217, Y: 17.605, Z: −25.603 and box dimension was set to X: 20, Y: 26, Z: 24. The binding affinities of the compounds with M^pro^ protein were calculated and ranked.

### 2.7. Protein Expression and Purification of SARS-CoV-2 M^pro^

The plasmid pET-28b-SARS-CoV-2-M^pro^ was a kind gift from Professor George Fu Gao from the Institute of Microbiology, Chinese Academy of Sciences. The expression plasmid was transformed into *E. coli* strain BL21 cells and then cultured in LB medium containing 50 μg/mL kanamycin in a shaking incubator at 37 °C. When the cells were grown to an OD_600_ of 0.6–0.8, 0.6 mM IPTG was added to the cell culture to induce the protein expression at 16 °C. After 18 h, the cells were harvested by centrifugation at 4000 rpm for 20 min at 4 °C. The cell pellets were washed twice by PBS, resuspended in lysis buffer (50 mM HEPES, 300 mM NaCl, 10 mM imidazole, pH 7.5), lysed by sonication on ice for 3 s ON time 5 s OFF time for 30 min of total time and then clarified by ultracentrifugation at 18,000 rpm at 4 °C for 40 min to remove debris. The supernatants were then purified by TALON metal affinity resin and washed with washing buffer (25 mM HEPES, 500 mM NaCl, pH 7.5) to remove unspecific binding proteins. The His-tagged M^pro^ was eluted by elution buffer (25 mM HEPES, 500 mM NaCl, 300 mM imidazole, pH 7.5). His-tagged SUMO protease (home-made) was added to remove the His-tag, His-tagged SUMO protease and uncleaved His-tag protein overnight at 4 °C. The M^pro^ was further purified by His60 Ni superflow resin. The quality of M^pro^ was checked by SDS-PAGE, and the concentration of M^pro^ was determined via a BCA Protein Assay Kit. The purified M^pro^ was stored in (10 mM Tris-HCl, 1 mM DTT, 1 mM EDTA, 10% glycerol, pH 7.5).

### 2.8. FRET-Based M^pro^ Enzyme Activity Inhibition Assay

Fluorescence resonance energy transfer (FRET)-based M^pro^ enzyme activity inhibition assay was conducted as follows. First, 5 μL serially diluted concentrations of candidate compounds were incubated with 35 μL 150 nM M^pro^ in Assay Buffer (10 mM Tris-Hcl, pH 7.5; 1 mM DTT; 1 mM EDTA; 0.01% Triton X-100) in a 96-well plate at room temperature for 30 min. This was followed with the adding of 10 μL 20 μM fluorogenic substrate (Dabcyl-KTSAVLQSGFRKME-Edans, P9733-5 mg, purchased from Beyotime) in Assay Buffer on ice, after which the plate was shaken for 1 min and then transferred to a 37 °C incubator for 30 min of incubation. Fluorescence signals (excitation wavelength at 340 nm and emission wavelength at 490 nm) were measured using a PerkinElmer Envision multimode plate reader. Experiments were performed in triplicate. Experimental data were plotted by GraphPad Prism 8.0.

## 3. Results

### 3.1. Dataset Preprocessing and Chemical Space Analysis

**Figure 1:**
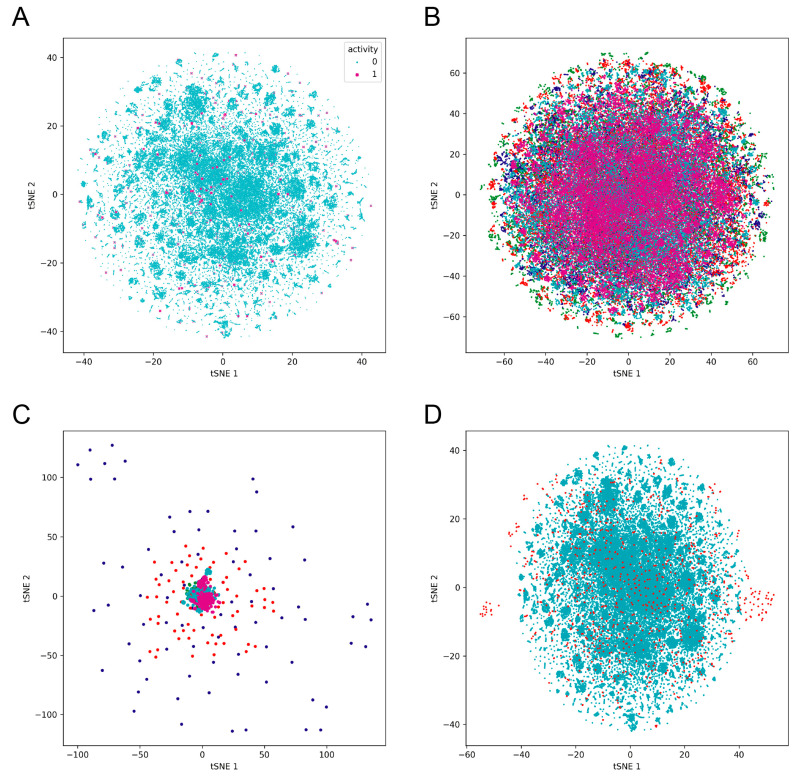
t-Distributed stochastic neighbor embedding (t-SNE) analysis of (**A**) active molecules (magenta) and inactive molecules (cyan) of the original dataset; (**B**) molecules in five subsets; (**C**) active molecules in five subsets; (**D**) molecules in original dataset (cyan) and the independent test dataset (red).

Because of the highly conserved sequence and the similar substrate binding site of M^pro^ between SARS-CoV-1 and SARS-CoV-2, the previously described inhibitors targeting SARS-CoV-1 M^pro^ could be used as templates for the design of novel inhibitors against SARS-CoV-2. Thus, the dataset used for fine-tuning was collected from PubChem (AID:1706) and from the literature, which contains 629 active molecules and 288,940 inactive molecules [29,50]. Structural relationships between active compounds and inactive compounds using t-SNE (t-distributed stochastic neighbor embedding) were calculated (Figure 1A). Analysis details were provided in the Supplementary Information. Data obtained from PubChem were the result of a QFRET-based biochemical high-throughput screening assay. Two inactive molecules were dropped due to long SMILES length, which is over 150. Scaffold-based 5-fold split was used to split the data. Due to the high imbalance of the lab dataset, data augmentation via a SMILES enumeration script was used to create more copies of active molecules. As shown in Table 1, different ratios of augmentation were conducted for later comparison to seek the optimum dataset size. To confirm the scaffold differences among the five-fold compounds, we also analyzed the structural relationships among the five-fold molecules using t-SNE (Figure 1B).

### 3.2. Performance of the Fine-Tuned Model

We used transfer learning to fine-tune a classification model for M^pro^ target bioactivity prediction. A pre-trained ChemBERTa model was downloaded from huggingface. To compare the performance of the classifier on different datasets, we calculated various evaluation scores using five-fold cross-validation. As shown in Table 2, the pre-trained model using augmented training data displayed better predictive ability on the validation dataset than no augmented data. An obvious improvement of evaluation scores was observed in all augmented datasets, especially in datasets with augmented active molecules 20 and 80 times. In addition, with augmented datasets, the pre-trained model for downstream task learning outperformed Graph Convolution Neural Network (GCNN) and baseline model Random Forest (RF).

To assess the model performance more realistically, we also evaluated on an external test dataset [47]. The external test dataset contains 880 fragments including 78 hits, which were screened through a combined mass spectrometry and X-ray approach against SARS-CoV-2 M^pro^. The structural diversity between the training and external datasets was also analyzed using t-SNE, as shown in Figure 1D. As shown in Table 3, a drop in performance on the external dataset was observed compared with the performance on the validation dataset, which was expected because no molecules in the test dataset were learned by the model before. The F1 score is one of the most meaningful metrics because it represents the harmonic mean of recall and precision. Datasets with 20 times more active molecules exhibited the highest f1 score of 0.34793, while GCNN and RF using the same training dataset only scored 0.0788 and 0.02025, respectively. Au_prc and au_roc were two other evaluation metrics for imbalanced data, while the former is more sensitive to the improvements of the positive class, which is a better indicator. In fine-tuned models, training datasets with 10 and 20 times more active molecules achieved similar au_prc scores, of 0.28671 and 0.28472, respectively, while the 80 times augmented datasets achieved a lower au_prc of 0.23152.

Having evaluated performances of various models and confirmed the advantages of data augmentation, we used the whole dataset as training input to compare the prediction abilities of transfer learning and a freely available classifier chemprop (http://chemprop.csail.mit.edu/ (accessed on 27 October 2021)) on this external test dataset. Chemprop could be used for molecular property prediction through a Message Passing Neural Network (MPNN), which works directly on a molecular graph [48]. Transfer learning with a 20 times augmented dataset achieved the highest au_prc of 0.34433, while the AUC-PR of chemprop was 0.19321. The f1 score of transfer learning using 20 times augmentation data was 0.41321, while that of chemprop was 0.19048 (Table 4).

### 3.3. Prediction of Bioactivities of Natural Compound and De Novo Generated Molecule Libraries

**Figure 2:**
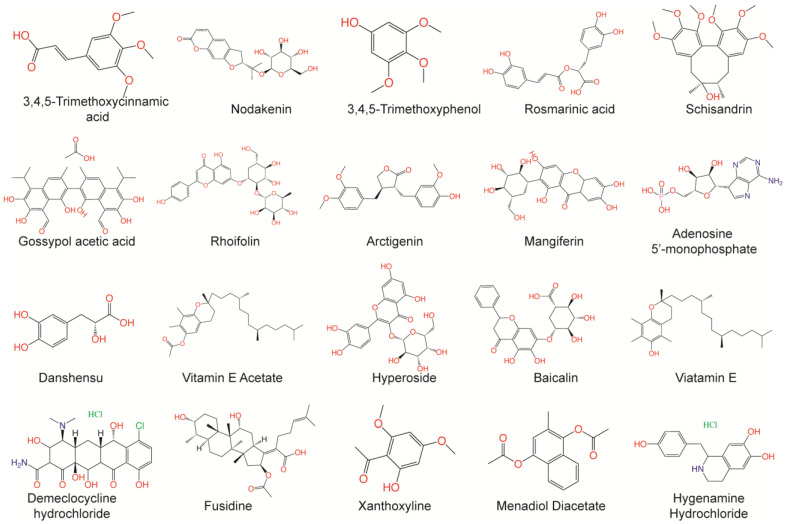
Top ranked 20 natural compounds screened by fine-tuned model. Rankings were from averages of five independent model predictions.

**Figure 3:**
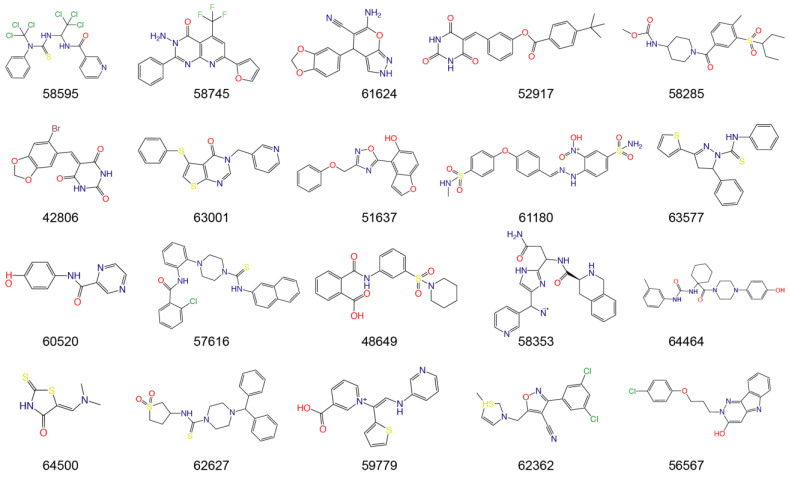
Top ranked 20 *de novo* generated molecules screened by fine-tuned model. Rankings were from averages of five independent model predictions.

The fine-tuned model using a 20 times augmented dataset was then used for making predictions of the Targetmol natural compound library and a *de novo* generated molecule library. Scoring ranks were the average results of five independent predictions. A total of 385 natural compounds and 66 *de novo* generated molecules were predicted as bioactive. The lists of predicted active compounds are provided in Tables S1 and S2. The top ranked 20 compounds from the natural compound library and 20 from the *de novo* generated molecule library are shown in Figure 2 and Figure 3, respectively.

### 3.4. Molecular Docking Screening

We next submitted all the predicted active compounds to docking simulation using AutoDock Vina (version1.2.0). Crystal structures of SARS-CoV-2 M^pro^ in complex with inhibitor PF-07321332 (PDB:7VH8) and N3 (PDB:6LU7) were both downloaded from the Protein Data Bank. PF-07321332 (Paxlovid) is an oral SARS-CoV-2 M^pro^ inhibitor developed by Pfizer and has shown positive responses in Phase III trials in combination with Ritonavir [51]. N3 is a covalent inhibitor of SARS-CoV-2 M^pro^ derived from the inhibitor targeting SARS-CoV-1 M^pro^ [15]. After calculating the binding affinities of the compounds with M^pro^, 46 compounds were selected for further binding pose analysis according to a cutoff score of −8.5 kcal/mol. After analysis of residue interactions in crystal structures of M^pro^ with PF-07321332 and N3, ligand interactions with F140 and E166 were considered critical for binding with M^pro^. Twelve molecules were finally confirmed as hits due to more than two H-bonds formed with residues F140 and E166. These hits include 10 natural compounds (T5429, T2727, T5497, T1035, T1609, T6S1529, T3149, T3S1612, TL0006) and two *de novo* generated molecules (58353 and 52917). The binding poses of these hit compounds with M^pro^ are displayed in Table 5.

### 3.5. PAINS Filtering

In the final round of the in silico analysis, we performed PAINS (pan assay interference compounds) filtering through a freely available web server FAF-Drugs4 to estimate potential molecules that may interfere with biological assays [49]. These compounds may display false positives in screening assays via a number of means and therefore represent poor choices for drug development [52]. We submitted all predicted active molecules to the server; 78 natural compounds and 5 *de novo* generated molecules were flagged as PAINS. For those natural compounds, among the top 20 predicted hits and 10 high-dock-scoring hits, T2765 (rosmarinic acid), T2730 (gossypol acetic acid), T3012 (mangiferin), T3227 (danshensu), T2844 (hyperoside), T2775 (baicalin), T3232 (higenamine hydrochloride), T5429 (theaflavin 3,3′-digallate), T2727 (salvianolic acid B), T6S1529 (1,5-Dicaffeoylquinic acid), T3149 (salvianolic acid C), TL0006 (chicoric acid) and T3242 (breviscapin) were flagged as PAINS. For those *de novo* generated molecules, among the top 20 predicted hits and two high dock-scoring hits, compound 52917, compound 42806, compound 64500 and compound 58353 were flagged as PAINS. However, virtual filters may not be perfect in identifying molecules that interfere with biological assays. Therefore, the judgement of PAINS should be taken with caution, and experimental confirmation is always necessary before any ‘problematic’ molecules are discarded.

### 3.6. In Vitro Binding Assay Validation

**Figure 4:**
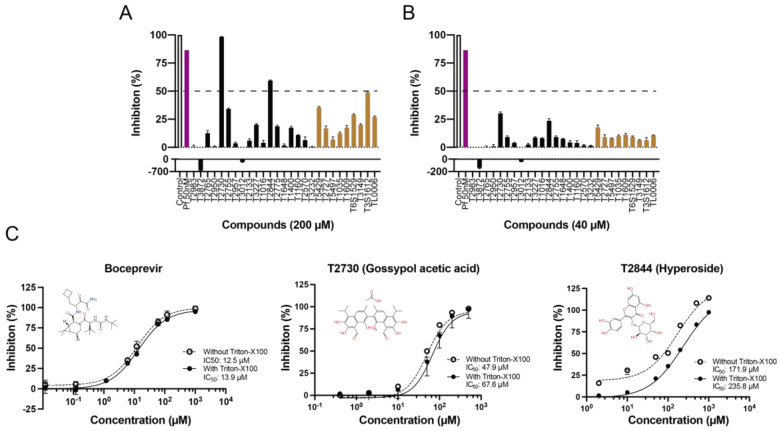
Inhibition of SARS-CoV-2 M^pro^. (**A**) Inhibition percentage of selected compounds at concentrations of 200 μM. (**B**) Inhibition percentage of selected compounds at concentrations of 40 μM. (**C**) Representative curves of Boceprevir, compound T2730 and T2844. All data are from at least three independent experiments and shown as mean ± SD.

In order to validate the in vitro binding activities of selected hits, we purchased 18 natural compounds from the top 20 scored active compounds predicted by deep learning and 9 selected natural compounds screened by molecular docking from Targetmol. PF-07321332 and Boceprevir were used as positive controls. These 27 compounds were tested by SARS-CoV-2 M^pro^ inhibition assay at concentrations of 200 μM and 40 μM. As shown in Figure 4A, except for PF-07321332, only compound T2730 (Gossypol acetic acid) and T2844 (Hyperoside) had over 50% inhibitory effects against M^pro^ catalytic activity at 200 μM, while all tested compounds exhibited less than 50% inhibitory effects at 40 μM. The IC50 values of compounds T2730 and T2844 were further determined in dose-dependent studies, which are 67.6 μM and 235.8 μM, respectively. Noteworthily, as many researchers have reported that some molecules self-associating into colloidal aggregates is one of the most common cause of non-specific inhibition [53,54], we added detergent triton X-100 in the experimental solvent; thus, the false positives caused by aggregate-based inhibition could be avoided. When treated with and without triton X-100, the inhibitory efficacies of the positive control Boceprevir and compound T2730 displayed no obvious differences within the experimental error, although a slight decrease in the inhibitory effects of T2844 when added with triton-X100 was observed. Gossypol acetic acid, a polyphenolic compound isolated form cottonseeds, has been reported to inhibit Bcl-2, Bcl-xL and Mcl-1 function and have antiproliferative effects on some cancer cells in vitro [55]. Hyperoside, a naturally occurring flavonoid compound isolated from Artemisia capillaris, shows myocardial protective, hepatoprotective, anti-redox and anti-inflammatory activities [56]. It is also a derivative of quercetin, which was predicted to potentially inhibit SARS-CoV-2 M^pro^ [57]. Recently, Dr. Souza’s group has demonstrated a biflavonoid (agathisflavone) and two flavonols (myricetin and fisetin) as non-competitive inhibitors of SARS-CoV-2 M^pro^, which indicated an interesting potential mode of action of these classes of compounds [58,59]. Further studies to deeper understand the mechanism of actions of these compounds are essential for chemical design to improve the activity profiles. Taken together, we have found that two natural compounds showed biological activity against M^pro^ in vitro.

## 4. Discussion

Artificial intelligence-aided drug design is becoming extensively used especially for emerging diseases because of its potential advantage in saving the time and cost of the drug discovery and development process. Here, we used a data augmentation method to boost transfer learning model performance in the fine-tuned bioactivity prediction task. The model outperformed GCNN, RF and chemprop. A natural compound library and a *de novo* generated molecule library were screened by this fast and efficient model. In combination with frequently used CADD techniques, such as molecular docking and PAINS-filtering, this method allowed us to select a group of 27 commercial available compounds for further experimental validation. Among these experimentally tested compounds, gossypol acetic acid and hyperoside displayed inhibitory effects against M^pro^ with IC50 values of 67.6 μM and 235.8 μM, respectively. Even though these two compounds displayed only micromolar potency, they still provided valuable scaffolds for further drug design in searching for treatment of COVID-19. Follow-up cellular assays and in vivo experiments are also essentially necessary to ensure the efficacy and safety of these compounds and more deeply understand the mechanism of actions. Overall, our results demonstrated the feasibility of finding potential candidate compounds using a deep learning method, and the experimental outcome suggested that these natural products may merit further biological studies of their potential ability in blocking SARS-CoV-2 infection.

## Figures and Tables

**Table 1:** Augmented dataset.

	Fold 1		Fold 2		Fold 3		Fold 4		Fold 5	
Label	1	0	1	0	1	0	1	0	1	0
Original	142	57,771	75	57,838	168	57,754	164	57,749	80	57,833
Augmentation_10	1420	57,771	750	57,838	1680	57,754	1640	57,749	800	57,833
Augmentation_20	2840	57,771	1500	57,838	3260	57,754	3280	57,749	1600	57,833
Augmentation_80	11,360	57,771	6000	57,838	13,440	57,754	13,120	57,749	6400	57,833

**Table 2:** Performance of fine-tuned model, GCNN model and RF on validation dataset.

Model		Transfer Learning			GCNN		Random Forest	
Dataset	Original	Active× 10	Active× 20	Active× 80	Active× 20	Active× 80	Active× 20	Active× 80
mcc	0.06931	0.88577	0.77580	0.97618	0.17995	0.26405	0.47148	0.46608
tp	1.4	1021.4	2294.6	9712	192	2160	948	3792
tn	57787.4	57757.8	57761.6	57755	57489.2	54236.6	57784.2	57783.8
fp	0	29.8	26	32.6	298.4	3551	3.4	3.8
fn	124.4	236.6	219.6	352	232.4	7904	1568	6272
auroc	0.52137	0.96239	0.98226	0.99226	0.76543	0.76389	0.77730	0.77897
auprc	0.03054	0.88366	0.95221	0.98621	0.3119	0.50636	0.52265	0.65047
recall	0.01532	0.80871	0.90753	0.96350	0.08885	0.19786	0.31199	0.31759
accuracy	0.99785	0.99550	0.98794	0.99436	0.95663	0.83353	0.97394	0.90750
precision	0.36667	0.97514	0.98818	0.99568	0.59492	0.84163	0.97671	0.99222
f1	0.02881	0.88391	0.94605	0.97931	0.11763	0.20417	0.38709	0.39592

**Table 3:** Performance of fine-tuned model, GCNN model and RF on an external dataset.

Model		Transfer Learning			GCNN		Random Forest	
Dataset	Original	Active× 10	Active× 20	Active× 80	Active× 20	Active× 80	Active× 20	Active× 80
mcc	0	0.30798	0.30973	0.26022	0.05526	0.08691	0.08652	0.08652
tp	0	16.6	22.6	26.2	4.8	11.8	0.8	0.8
tn	802	789.8	774	746.2	787.8	754.2	802	802
fp	0	12.2	28	55.8	14.2	47.8	0	0
fn	78	61.4	55.4	51.8	73.2	66.2	77.2	77.2
auroc	0.50025	0.66905	0.67788	0.68109	0.66972	0.68249	0.68298	0.71836
auprc	0.08868	0.28671	0.28427	0.23152	0.20616	0.23784	0.35956	0.40239
recall	0	0.21282	0.28974	0.39990	0.06154	0.15128	0.01026	0.01026
accuracy	0.72909	0.91636	0.90523	0.87773	0.90068	0.87045	0.91227	0.91227
precision	0	0.58623	0.44416	0.31989	0.13992	0.23694	0.8	0.8
f1	0	0.29778	0.34973	0.32647	0.07880	0.10446	0.02025	0.02025

**Table 4:** Performance of fine-tuned model and Chemprop on an external dataset.

Model	Input	Mcc	Auroc	Auprc	Recall	Accuracy	Precision	f1
Transfer Learning	Active × 20	0.37804	0.68186	0.34433	0.34359	0.91341	0.51978	0.41321
	Active × 80	0.29978	0.68306	0.26118	0.34359	0.89091	0.37632	0.35833
Chemprop	original	0.17636	0.68152	0.19321	0.12821	0.90341	0.37037	0.19048

**Table 5:** Summary of selected molecules screened against SARS-CoV-2 M^pro^ with their structures, binding affinities and interactive residues.

IDs	Name	Source	Structure	Docking Score (kcal/mol)	M^pro^ Residues Interacting with Molecules through H-Bond and Other Types
PF-07321332	-	-	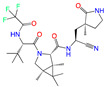	−9.2	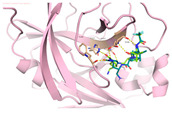
T5429	Theaflavin 3,3′-digallate	Black tea	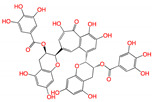	−10.4	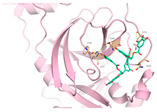
T2727	Salvianolic acid B	Slvia miltiorrhiza	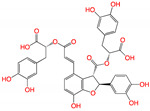	−9.2	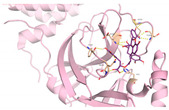
T5497	AMAROGENTIN	Gentiana scabra	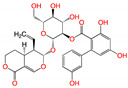	−8.9	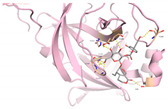
T1035	Hesperidin	Citrus sinensis	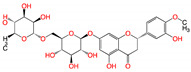	−8.8	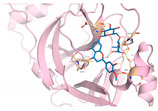
T1609	NAD+	Punica granatum	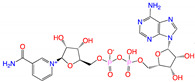	−8.8	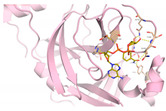
T6S1529	1,5-Dicaffeoylquinic acid	Lonicera japonica	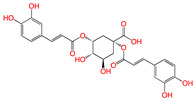	−8.8	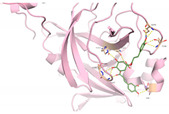
T3149	Salvianolic Acid C	Slvia miltiorrhiza	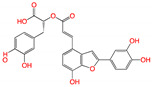	−8.7	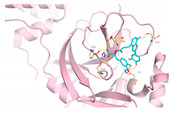
T3S1612	Kuwanon G	Morus alba	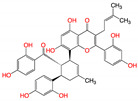	−8.7	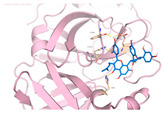
TL0006	Chicoric Acid	Cichorium intybus	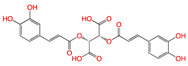	−8.6	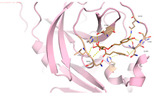
T3242	Breviscapin	Erigeron	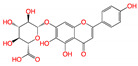	−8.5	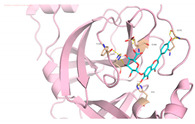
58353	-	-	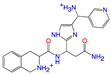	−8.7	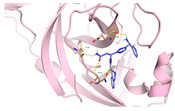
52917	-	-	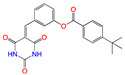	−8.6	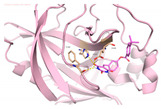

## Data Availability

The pre-trained model used for transfer learning can be freely downloaded from huggingface (https://huggingface.co/seyonec/ChemBERTa-zinc-base-v1 (accessed on 15 June 2021)). AutoDock Vina (version 1.2.0) used for molecular docking can be downloaded from GitHub repository (https://github.com/ccsb-scripps/AutoDock-Vina (accessed on 11 November 2021)). The web server FAF-Drugs4 used for PAINS filtering is publicly available at https://fafdrugs4.rpbs.univ-paris-diderot.fr/. All relevant data are shown in figures, tables and Supporting Materials. The ChemBERTa model predicted active natural compound and *de novo* generated compound information are listed in Table S1 and Table S2, respectively. Molecular docking scores are provided in Table S3.

## Supplementary Materials

The following supporting information can be downloaded at: https://www.mdpi.com/article/10.3390/v15040891/s1, the Fine-tuned model prediction results are provided in Tables S1 and S2 (.xls). Table S1: Predicted_Active_Natural_Compounds; Table S2: Predicted_Active_De_novo_Generated_Compounds; Table S3: Docking_scores.
